# Assembly of MSCs into a spheroid configuration increases poly(I:C)-mediated TLR3 activation and the immunomodulatory potential of MSCs for alleviating murine colitis

**DOI:** 10.1186/s13287-025-04297-3

**Published:** 2025-04-12

**Authors:** Chao-Ting Ho, Ying-Chi Kao, Yueh-Ming Shyu, I-Ching Wang, Qiao-Xuan Liu, Shao-Wen Liu, Shih-Chen Huang, Han Chiu, Li-Wen Hsu, Tzu-Sheng Hsu, Wan-Chen Hsieh, Chieh-Cheng Huang

**Affiliations:** 1 Institute of Biomedical Engineering, National Tsing Hua University, Hsinchu, Taiwan; 2 Institute of Molecular and Cellular Biology, College of Life Sciences and Medicine, National Tsing Hua University, Hsinchu, Taiwan; 3 Bioresource Collection and Research Center, Food Industry Research and Development Institute, Hsinchu , Taiwan

**Keywords:** Inflammatory bowel disease, Mesenchymal stem cell, Cell spheroid, Toll-like receptor, Immunomodulation

## Abstract

**Background:**

Inflammatory bowel disease (IBD) is associated with significant clinical challenges due to the limitations of current therapeutic approaches. Mesenchymal stem cell (MSC)-based therapies have shown promise in alleviating IBD owing to their potent immunomodulatory properties. However, the therapeutic efficacy of these cells remains suboptimal, primarily due to the harsh peritoneal microenvironment, which compromises MSC viability and functional capacity after transplantation.

**Methods:**

To address these limitations, this study aimed to improve MSC engraftment and functionality by assembling MSCs into three-dimensional (3D) spheroids and priming them with the Toll-like receptor 3 (TLR3) agonist polyinosinic-polycytidylic acid (poly(I:C)). Their potential for treating IBD was evaluated using male C57BL/6 mice with dextran sulfate sodium-induced colitis.

**Results:**

While 3D spheroid formation alone upregulated TLR3 expression and increased MSC survival under oxidative stress, poly(I:C) priming had a pronounced synergistic effect, significantly increasing MSC-mediated splenocyte modulation and oxidative stress resistance. In a murine colitis model, compared with unprimed spheroids or MSC suspensions, poly(I:C)-primed MSC spheroids administered intraperitoneally exhibited increased survival and therapeutic efficacy, effectively alleviating colitis symptoms, reducing colonic inflammation, and promoting tissue recovery.

**Conclusion:**

Collectively, these findings highlight the synergistic benefits of combining 3D spheroid assembly with TLR3 activation as an innovative strategy to improve the therapeutic efficacy of MSC-based treatments for IBD and other inflammatory diseases by increasing post-engraftment cell survival and immunomodulatory capacity.

**Supplementary Information:**

The online version contains supplementary material available at 10.1186/s13287-025-04297-3.

## Background

Inflammatory bowel disease (IBD), encompassing Crohn’s disease and ulcerative colitis, arises from a loss of immune tolerance, leading to dysregulated immune responses and chronic mucosal inflammation [1]. Overactivation of antigen-presenting cells, such as dendritic cells and macrophages, promotes excessive CD4+ T cell activation and polarization toward proinflammatory phenotypes, perpetuating inflammation [2]. Moreover, neutrophil infiltration intensifies tissue damage, further promoting disease progression [2]. Additionally, oxidative stress not only accumulates in the inflamed mucosa [3] but also extends into deeper intestinal layers and systemic circulation [4], activating proinflammatory signaling cascades that disrupt tissue homeostasis and exacerbate colitis. While regulatory T cells (Tregs) normally suppress immune activation and maintain tolerance, in IBD, they become functionally impaired or outnumbered, shifting the immune balance toward chronic inflammation [1, 2, 5]. The resulting persistent inflammation damages the small intestine and colonic mucosa, contributing to the rising prevalence of IBD and imposing substantial health and economic burdens worldwide [6].

Conventional therapies for IBD focus primarily on managing symptoms through immune suppression. However, the efficacy of these treatments is limited by their severe side effects and associated risk of opportunistic infections; furthermore, these treatments lack effectiveness in refractory cases [3, 7–10]. Consequently, these limitations hinder the effective control of long-term inflammation, significantly impairing patients’ quality of life and underscoring the urgent need for more innovative and effective treatment strategies.

Recent advances have highlighted the potential of cell-based therapies as promising approaches for treating IBD [7]. Among these therapies, those based on mesenchymal stem cells (MSCs) are particularly notable because of the ease of collection, tolerogenic phenotype, immunomodulatory ability, and pro-regenerative capacity of MSCs [7, 11–14]. MSCs can respond to inflammatory stimuli by releasing various factors that modulate the behavior of immune cells, alleviating inflammation in IBD tissues and improving tissue repair [7, 11–15].

Despite the promising attributes of MSC-based therapies for IBD, significant hurdles persist, notably, the heterogeneity in therapeutic efficacy due to poor engraftment efficiency [16, 17]. Systemic injections often result in MSCs becoming trapped in organs unrelated to the target inflamed areas, thereby diminishing the impact of MSC-based therapies [18]. Alternatively, while intraperitoneal administration generally leads to better results than systemic injections, the survival rate and therapeutic potential of MSCs within the harsh environments of IBD tissues, which are characterized by inflammation and elevated oxidative stress, remain critically low [12, 16]. In particular, reactive oxygen species-induced oxidative stress disrupts MSC adhesion, induces apoptosis, and impairs MSC immunomodulatory capacity post-transplantation [12, 16, 19, 20]. Consequently, the therapeutic success of MSC-based interventions depends on both efficient engraftment and sustained functionality [17, 21]. Therefore, the development of strategies that increase the engraftment efficiency and improve the functional capabilities of MSCs under these challenging conditions is crucial [12, 16, 22, 23].

To address these challenges, various preconditioning or priming strategies have been adopted in recent decades before transplantation to increase MSC survival and optimize therapeutic outcomes [24]. For example, research conducted by our team [25–30] and others [31–35] has shown that culturing MSCs in a three-dimensional (3D) multicellular spheroid format significantly increases their post-engraftment viability in harsh microenvironments and improves their therapeutic functions, particularly in terms of paracrine secretion. Despite these advances, it remains unclear whether these enhanced capabilities translate directly into improved efficacy for IBD treatment, necessitating further investigation.

In this study, we preassembled MSCs into 3D spheroids and administered them intraperitoneally to mice with dextran sulfate sodium (DSS)-induced colitis, with the aim of reducing tissue inflammation. The DSS-induced mouse colitis model was selected for its well-documented ability to replicate key pathological features of human colitis, such as epithelial barrier disruption and immune activation, while offering a reproducible and well-controlled experimental platform for studying IBD pathogenesis [36, 37]. To further increase the immunomodulatory capabilities of these MSC spheroids, we employed polyinosinic-polycytidylic acid (poly(I:C)), a known agonist of Toll-like receptor 3 (TLR3). Poly(I:C) has been shown to enhance the paracrine and immunomodulatory activities of MSCs [38–40], significantly amplifying their therapeutic potency in murine IBD models [16]. However, existing research has focused predominantly on the effects of poly(I:C) priming on MSCs cultivated in a two-dimensional (2D) monolayer, a configuration that drastically differs from the 3D spheroid environment. This discrepancy underscores the need for further research to evaluate whether the immunomodulatory benefits of poly(I:C) are effectively retained or increased in the 3D MSC setup.

In pursuit of this, our study specifically aimed to improve IBD treatment outcomes by leveraging both the 3D spheroid structure and the activation of TLR3 signaling through poly(I:C) priming. Our in vitro results demonstrated a synergistic effect, where TLR3 activation by poly(I:C) not only persisted but was also increased in the 3D spheroid arrangement. This improvement significantly increased the viability, paracrine function, and immunomodulatory capacity of the MSCs under adverse conditions. Furthermore, the therapeutic application of these poly(I:C)-primed MSC spheroids in vivo effectively suppressed colitis and promoted intestinal recovery in an IBD mouse model. Notably, these outcomes were superior to those observed in animals treated with unprimed MSC spheroids. These findings highlight the synergistic impact of poly(I:C) priming and the 3D spheroid model, highlighting a significant advancement in the deployment of MSCs as immunomodulatory agents for managing inflammation and facilitating tissue regeneration.

## Methods

### Preparation of poly(I: C)-primed MSC spheroids

Human umbilical cord blood-derived MSCs (BCRC #60605) obtained from the Food Industry Research and Development Institute (Hsinchu, Taiwan) [41] were previously characterized for their surface markers and multilineage differentiation potential [42] and were subsequently transfected with a lentiviral vector to drive the expression of luciferase [25]. The MSCs were cultured in α-minimal essential medium (Thermo Fisher Scientific, Waltham, MA, USA) supplemented with 20% fetal bovine serum (FBS; Corning, Corning, NY, USA), 4 ng/mL basic fibroblast growth factor (PeproTech, Rocky Hill, NJ, USA), and 30 µg/mL hygromycin B (Thermo Fisher Scientific). To facilitate the formation of cell spheroids, 20,000 MSCs were suspended in culture medium with or without 10 µg/mL poly(I:C) [15, 43], transferred into a 96-well plate coated with 12% methylcellulose to create a nonattachable surface, and cultivated for 24 h. The resulting MSC spheroids were examined under a phase-contrast microscope, and the photomicrographs were analyzed with ImageJ software (National Institutes of Health, Bethesda, MD, USA) for measurement of the size and circularity of the spheroids.

### RNA isolation and real-time quantitative polymerase chain reaction (qPCR)

To evaluate the impact of 3D spheroid formation or poly(I:C) priming on the mRNA expression of target genes, total RNA was extracted from the test cells with the TOOLSmart RNA Extractor (Biotools; New Taipei City, Taiwan). The extracted RNA was then reverse transcribed in accordance with the instructions provided in the High-Capacity cDNA Reverse Transcription Kit (Thermo Fisher Scientific). The resulting cDNA was analyzed through qPCR with Genious 2X SYBR Green Fast qPCR Mix (ABclonal; Woburn, MA, USA) on a QuantStudio 3 Real-Time PCR System (Thermo Fisher Scientific). The mRNA expression levels of the target genes were quantified and normalized to those of ribosomal protein L13a (*RPL13A*) with the primers specified in Table S1.

### Western blot analysis

In addition, the test cells were lysed with RIPA buffer (Bio Basic; Amherst, NY, USA) supplemented with a protease inhibitor cocktail (Sigma‒Aldrich; St. Louis, MO, USA) [44]. The lysates were subjected to sodium dodecyl sulfate‒polyacrylamide gel electrophoresis on a 10% acrylamide gel (Bio-Rad Laboratories, Hercules, CA) at 80 V. The proteins were transferred to a polyvinylidene difluoride membrane (Cytiva; Marlborough, MA) at 200 mA for 2.5 h. The membrane was blocked by incubation in blocking buffer containing 5% skim milk for 1 h. Subsequently, the membranes were incubated overnight at 4 °C with shaking, and primary antibodies against β-actin (Abcam), cyclooxygenase-2 (COX-2; GeneTex), heme oxygenase-1 (HO-1; GeneTex), or indoleamine 2,3-dioxygenase-1 (IDO-1; GeneTex) were used. The next day, the membranes were incubated with horseradish peroxidase-conjugated secondary antibodies (GeneTex). Protein signals were detected *via* Amersham ECL Select Western Blotting Detection Reagent (Cytiva).

### Preparation and characterization of conditioned medium (CM) derived from poly(I:C)-primed MSC spheroids

MSC spheroids, with or without poly(I:C) priming, were collected and seeded into a 6-well culture plate (25 spheroids or 5 × 10^5^ MSCs per well) and then incubated overnight to allow attachment. The following day, the culture medium was removed and replaced with 2 mL of fresh medium, and the cultures were incubated for an additional 48 h. The CM was then collected and centrifuged at 1500 *× g* for 10 min to remove cellular debris. To assess changes in the secretome content of MSC spheroids induced by poly(I:C) priming, a human cytokine antibody array (ab193656; Abcam) was used to measure the relative levels of 120 cytokines following the manufacturer’s instructions [45]. The signal intensity of each cytokine spot was quantified *via* ImageJ software and normalized to the corresponding positive control spots. Alternatively, the prostaglandin E2 (PGE2) level in the collected CM was determined with an enzyme-linked immunosorbent assay (ELISA) kit (Cayman; Ann Arbor, MI, USA).

### Evaluation of the impact of poly(I:C) priming on the immunomodulatory potential of MSC spheroids in primary mouse splenocytes

Animal protocols were reviewed and approved by the Institutional Animal Care and Utilization Committee of National Tsing Hua University (IACUC Approval No. 111056), adhering to the 2018 Guidelines for the Care and Use of Laboratory Animals issued by the Council of Agriculture, Executive Yuan, Taiwan, and the Animal Research: Reporting of In Vivo Experiments (ARRIVE) guideline 2.0. Primary mouse splenocytes were isolated by euthanizing C57BL/6 mice (aged eight to ten weeks; BioLasco, Taipei, Taiwan) *via* CO_2_ inhalation, and their spleens were collected in RPMI 1640 medium supplemented with 5% FBS. The spleens were then mechanically dissociated with a 70-µm cell strainer and a 1-mL syringe plunger, followed by thorough rinsing with the same medium. After centrifugation, red blood cells were lysed with ACK lysis buffer (Thermo Fisher Scientific), and the splenocytes were then pelleted, resuspended in complete RPMI 1640 medium, and filtered through a 40-µm cell strainer. Viable splenocytes were counted *via* trypan blue exclusion and subsequently used for further experiments.

To evaluate regulatory T (Treg) cell differentiation, freshly isolated splenocytes were activated for 24 h with plate-bound anti-CD3 (10 µg/mL) and anti-CD28 (1 µg/mL) antibodies (Bio X Cell; Lebanon, NH, USA) in the presence of recombinant IL-2 (1 ng/mL; BioLegend; San Diego, CA, USA). After activation, the cells were cultured for 5 days in differentiation medium consisting of RPMI 1640, 10% FBS, 1 ng/mL IL-2, and 5 ng/mL transforming growth factor (TGF)-β (PeproTech), along with CM derived from MSC spheroids, either with or without poly(I:C) priming.

Following the treatment period, the splenocytes were stained for viability and surface markers, including CD4, CD8, CD25, and CD69. For the intracellular staining of Foxp3, the cells were fixed and permeabilized with the Foxp3/Transcription Factor Staining Buffer Set (eBioscience; San Diego, CA, USA), followed by incubation with an anti-Foxp3 antibody (eBioscience) for 1 h. After being washed twice with permeabilization buffer, the cells were analyzed with a CytoFlex analyzer (Beckman Coulter; Brea, CA, USA) and processed with FlowJo software (v10.5.3; FlowJo).

### Evaluation of the impact of poly(I:C) priming on MSC survival under oxidative stress

To assess whether poly(I:C) priming can improve the survival of MSCs in hostile microenvironments, 2 × 10^5^ MSCs were prepared as single-cell suspensions or spheroids with or without poly(I:C) priming. These cells were then collected and transferred into the wells of a 6-well culture plate supplemented with culture medium containing 100 µM hydrogen peroxide (H_2_O_2_) to simulate the oxidative stress encountered during cell delivery [3, 10, 46]. After 24 h, photomicrographs of the cultivated cells were captured with a phase-contrast microscope. Additionally, cell viability was determined with a cell counting kit (CCK)-8 assay [47, 48].

### Mouse IBD model

Male C57BL/6 mice (BioLasco), aged six to eight weeks, were housed at the Laboratory Animal Center of National Tsing Hua University under specific pathogen-free conditions, with an ambient temperature of 22 °C and a 12-h light/12-h dark cycle. A total of 36 mice were divided into six groups (*n* = 6 animals per group) using the random number table method. To induce colitis, 2.5% DSS (MP Biomedicals, Santa Ana, CA, USA) was administered in the drinking water from day 0 to day 5. MSCs, either as suspensions or spheroids and with or without poly(I:C) priming, were prepared with a total of 1 × 10^6^ cells suspended in 300 µL of saline and injected intraperitoneally on days 1 and 3 under anesthesia with 3% isoflurane. Mice that were not exposed to DSS, along with those that received DSS but were not administered MSCs, served as the control groups. Successful induction of the colitis model was determined by significant weight loss within five days of DSS administration initiation.

To assess the viability of the transplanted MSCs, 100 µL of Nano-Glo Live Cell Reagent (Promega, Madison, WI, USA) was administered intraperitoneally to the animals on days 4, 7, and 14. The animals were anesthetized with 1–3% isoflurane, and bioluminescence was measured 20 min post-injection with a Xenogen IVIS 200 System (Revvity, Waltham, MA, USA). To assess the therapeutic efficacy of MSC administration, the body weights of the test animals were monitored daily until day 17.

In a separate cohort, 24 mice were randomly assigned to four groups (*n* = 6 animals per group) to undergo DSS exposure and receive MSC spheroids, with or without poly(I: C) priming. Control groups included mice that were not exposed to DSS and those that received DSS but no MSC treatment. The mice were euthanized on day 9 *via* CO_2_ inhalation to collect colon tissue. After measuring its length, the tissue was then fixed in phosphate-buffered formalin, embedded in paraffin, sectioned, and stained with hematoxylin and eosin. For the evaluation of proinflammatory cytokine expression in colon tissue, the samples were homogenized, and total RNA was extracted for qPCR. The mRNA expression levels of the target genes were quantified and normalized to those of glyceraldehyde-3-phosphate dehydrogenase (*Gapdh*) with the primers specified in Table S1.

### Statistical analysis

Statistical analyses were conducted with GraphPad Prism software (version 10.2.3; San Diego, CA, USA). For comparisons between two groups, Student’s *t* test was employed. For comparisons among three or more groups, one-way ANOVA followed by Tukey’s *post hoc* correction was used. The results are expressed as the means ± standard deviations. A *p* value of less than 0.05 was considered statistically significant.

## Results

### Assembly of MSCs into 3D spheroids upregulates TLR3 expression

**Fig. 1:**
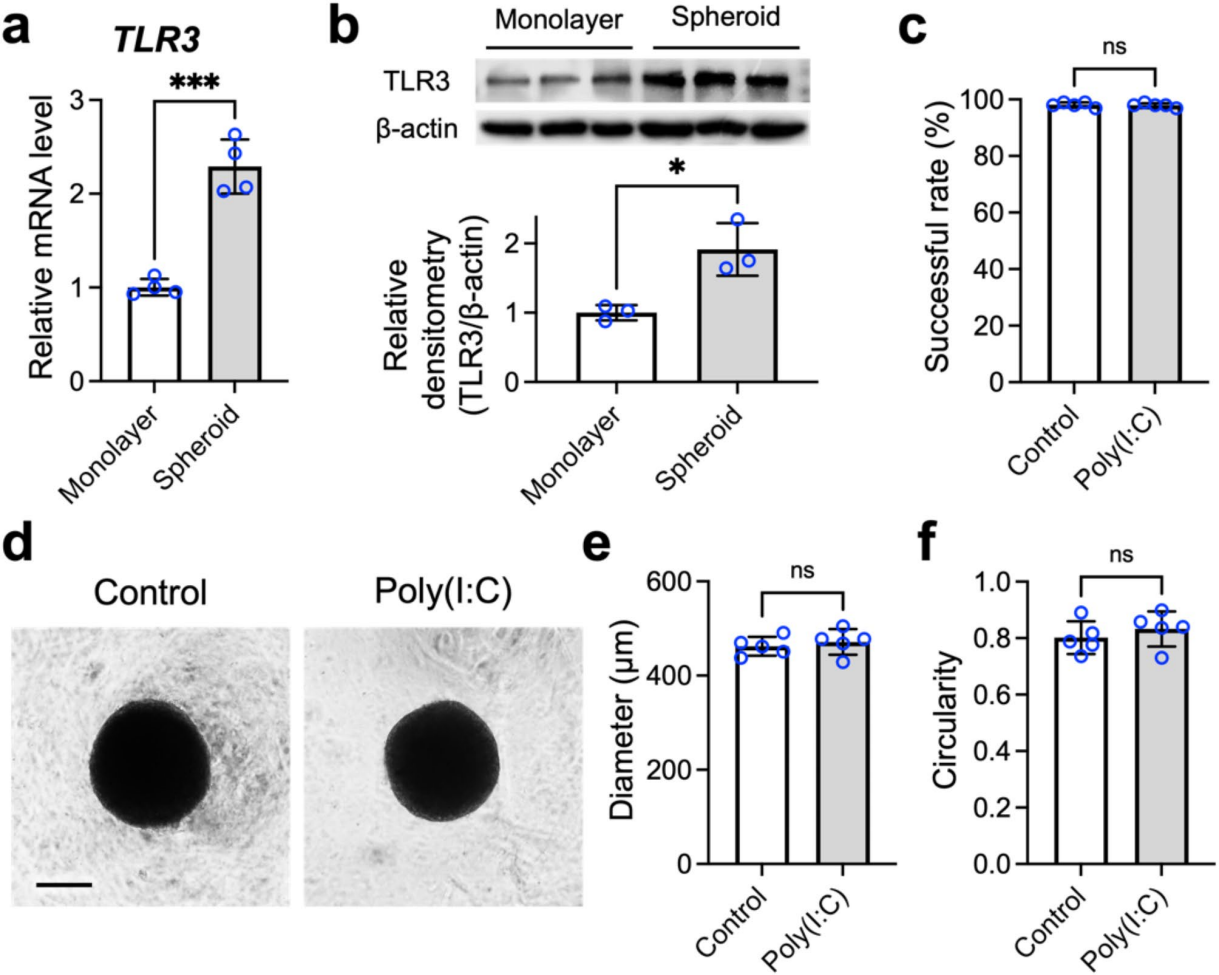
The assembly of mesenchymal stem cells (MSCs) into three-dimensional (3D) spheroids upregulates Toll-like receptor 3 (TLR3) expression. (**a**) Effects of spheroid formation on TLR3 mRNA (*n* = 4) and (**b**) protein levels (*n* = 3) in MSCs, as determined by real-time quantitative polymerase chain reaction (qPCR) and Western blotting, respectively. Full-length blots are presented in Supplementary Fig. 1a. (**c**) The impact of polyinosinic-polycytidylic acid (poly(I:C)) treatment on MSC spheroids was assessed by evaluating the spheroid formation success rate and their (**d**) gross morphology, (**e**) size, and (**f**) circularity (*n* = 5 batches of MSC spheroids). Scale bar, 200 μm. The data are represented as the means ± standard deviations (SDs). Student’s *t* test was used to determine the *p* values. *p < 0.05; ***p < 0.05; ns, not significant

To facilitate cellular assembly, the MSCs were suspended in culture medium and transferred to a culture plate featuring a nonadhesive surface established with a methylcellulose hydrogel, following our previously established methodology [25–30, 44, 49]. Before priming the cells with poly(I:C), we first explored the impact of spheroid formation on TLR3 expression in MSCs. Our qPCR data indicated that the assembly of MSCs into 3D spheroids resulted in the upregulation of *TLR3* expression (2.3-fold vs. monolayered MSCs; *p* < 0.005; Fig. 1a). This finding was further corroborated by our immunoblotting results, which showed a significant increase in TLR3 levels upon MSC assembly into 3D spheroids (1.9-fold vs. monolayered MSCs; *p* < 0.05; Fig. 1b). These findings suggest that the formation of 3D spheroids increases TLR3 expression by MSCs and potentially increases their sensitivity to poly(I:C).

Next, before assessing the impact of poly(I:C) supplementation on the therapeutic efficacy of MSC spheroids, we examined its influence on spheroid formation. The overall success rates for MSC spheroid formation were comparable between the groups investigated (98.1 ± 0.9% vs. 97.9 ± 0.7%; *p* > 0.05; Fig. 1c), indicating that poly(I:C) supplementation did not interfere with the assembly of MSCs into spheroids. Furthermore, representative phase-contrast images (Fig. 1d) revealed that the morphology of the MSC spheroids treated with poly(I:C) was similar to that of the untreated spheroids, with comparable diameters (462.1 ± 19.9 μm vs. 471.5 ± 27.4 μm; *p* > 0.05; Fig. 1e) and circularity (0.801 ± 0.058 vs. 0.833 ± 0.062; *p* > 0.05; Fig. 1f). These findings suggest that the application of poly(I: C) does not alter the spheroid formation behavior of MSCs.

### Assembly of MSCs into 3D spheroids and application of poly(I:C) synergistically upregulate the expression of immunomodulation-relevant molecules

We next investigated the effect of poly(I: C) on the mRNA expression of specific genes related to the immunomodulatory potential of MSC spheroids. Herein, the mRNA expression levels of *PTGS2* and *IDO1*, which encode COX-2 and IDO-1, respectively, were evaluated *via* qPCR. These genes are regulated by TLR3 signaling and produce the potent immunomodulators PGE2 and kynurenine [31, 50]. Additionally, the expression of *TNFAIP6* (encoding tumor necrosis factor-stimulated gene-6), *IL1RN* (encoding interleukin-1 receptor antagonist; IL-1ra), *HMOX1* (encoding HO-1), *IL4* and *IL10*, was also assessed. MSCs grown as 2D monolayers served as controls to determine the impact on cell culture dimensions.

**Fig. 2:**
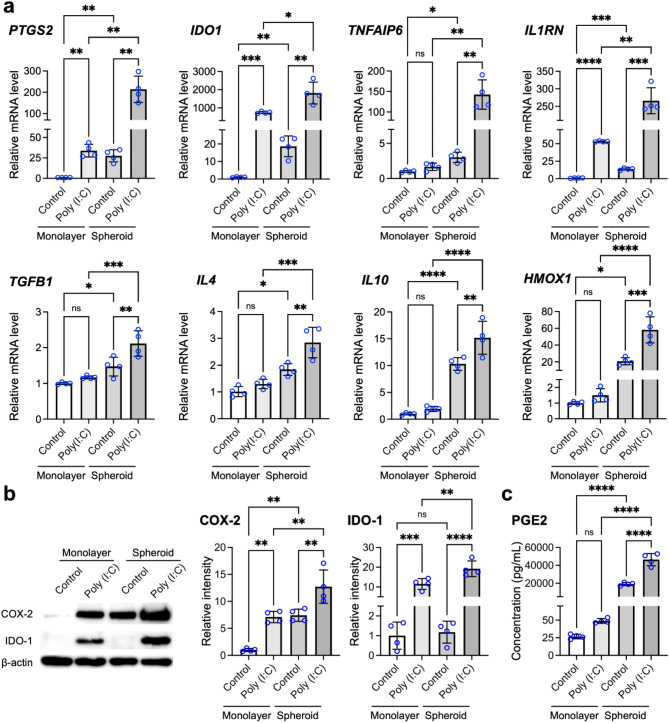
The assembly of MSCs into 3D spheroids and the application of poly(I:C) synergistically upregulate the expression of immunomodulation-related molecules. (**a**) Effects of spheroid formation and poly(I:C) treatment on the mRNA (*n* = 4) and (**b**) protein levels (*n* = 4) of TLR3 downstream effectors, as determined by qPCR and Western blotting, respectively. Full-length blots are presented in Supplementary Fig. 1b. (**c**) Prostaglandin E2 (PGE2) secretion by MSCs into the culture medium, as measured by an enzyme-linked immunosorbent assay (*n* = 4). The data are represented as the means ± SDs. Analysis of variance (ANOVA) followed by Tukey’s test was used to determine the *p* values. *p < 0.05; **p < 0.01; ***p < 0.005; ****p < 0.001; ns, not significant

Compared with monolayer-grown cells, MSC spheroids without poly(I:C) treatment showed significant increases in the mRNA expression level of *PTGS2* (27.6-fold; *p* < 0.01), *IDO1* (18.6-fold; *p* < 0.01), *TNFAIP6* (3.0-fold; *p* < 0.05), *IL1RN* (13.8-fold; *p* < 0.005), *TGFB1* (1.5-fold; *p* < 0.05), *IL4* (1.8-fold; *p* < 0.05), *IL10* (10.3-fold; *p* < 0.001) and *HMOX1* (20.7-fold; *p* < 0.05; Fig. 2a), suggesting that spheroid assembly alone could promote the immunomodulatory potential of MSCs. Furthermore, poly(I:C) treatment upregulated the expression of these genes in both monolayer and 3D spheroid configurations. In MSC spheroids, poly(I:C) treatment led to increases of 7.7-fold for *PTGS2* (*p* < 0.01), 97.3-fold for *IDO1* (*p* < 0.01), 47.2-fold for *TNFAIP6* (*p* < 0.01), 19.3-fold for *IL1RN* (*p* < 0.005), 1.4-fold for *TGFB1* (*p* < 0.01), 1.5-fold for *IL4* (*p* < 0.01), 1.5-fold for *IL10* (*p* < 0.01), and 2.8-fold for *HMOX1* (*p* < 0.005) compared with those in untreated spheroids (Fig. 2a), indicating that poly(I:C)-driven TLR3 activation might further increase the immunomodulatory potential of MSC spheroids. In summary, compared with untreated monolayer cells, MSCs assembled as 3D spheroids with poly(I:C) treatment substantially elevated mRNA expression levels of *PTGS2* (213.7-fold; *p* < 0.01), *IDO1* (1813.6-fold; *p* < 0.01), *TNFAIP6* (266.1-fold; *p* < 0.01), *IL1RN* (13.8-fold; *p* < 0.005), *TGFB1* (2.1-fold; *p* < 0.001), *IL4* (2.8-fold; *p* < 0.001), *IL10* (15.2-fold; *p* < 0.001), and *HMOX1* (58.2-fold; *p* < 0.001; Fig. 2a).

Our immunoblot results further corroborated the upregulation of *PTGS2* and *IDO1* genes observed at mRNA expression levels. We observed a significant increase in the protein expression of COX-2 (12.7-fold vs. untreated monolayer MSCs, *p* < 0.001; 1.7-fold vs. untreated MSC spheroids, *p* < 0.01) and IDO-1 (19.2-fold vs. untreated monolayer MSCs, *p* < 0.001; 16.3-fold vs. untreated MSC spheroids, *p* < 0.001; Fig. 2b). Additionally, increased secretion of PGE2, generated by the enzyme COX-2, was observed in the (CM) of poly(I:C)-primed MSC spheroids (46,352.1 pg/mL) compared with that in untreated monolayer MSCs (26.6 pg/mL; *p* < 0.001) and untreated MSC spheroids (19,111.1 pg/mL; *p* < 0.001; Fig. 2c), as determined by ELISA. Collectively, our experimental results demonstrate that assembling MSCs into 3D spheroids and applying poly(I:C) might be an effective combinational strategy for significantly increasing the expression of immunomodulation-related molecules by MSCs.

### Poly(I:C) priming increases the paracrine activity of MSC spheroids

After examining the expression of enzymes and molecules vital for the immunomodulatory capability of MSCs, we focused on observing changes in the paracrine profile of MSC spheroids induced by poly(I:C). To achieve this goal, we utilized cytokine antibody arrays to analyze the CM (which represents the secretome) of MSC spheroids with and without poly(I:C) treatment.

**Fig. 3:**
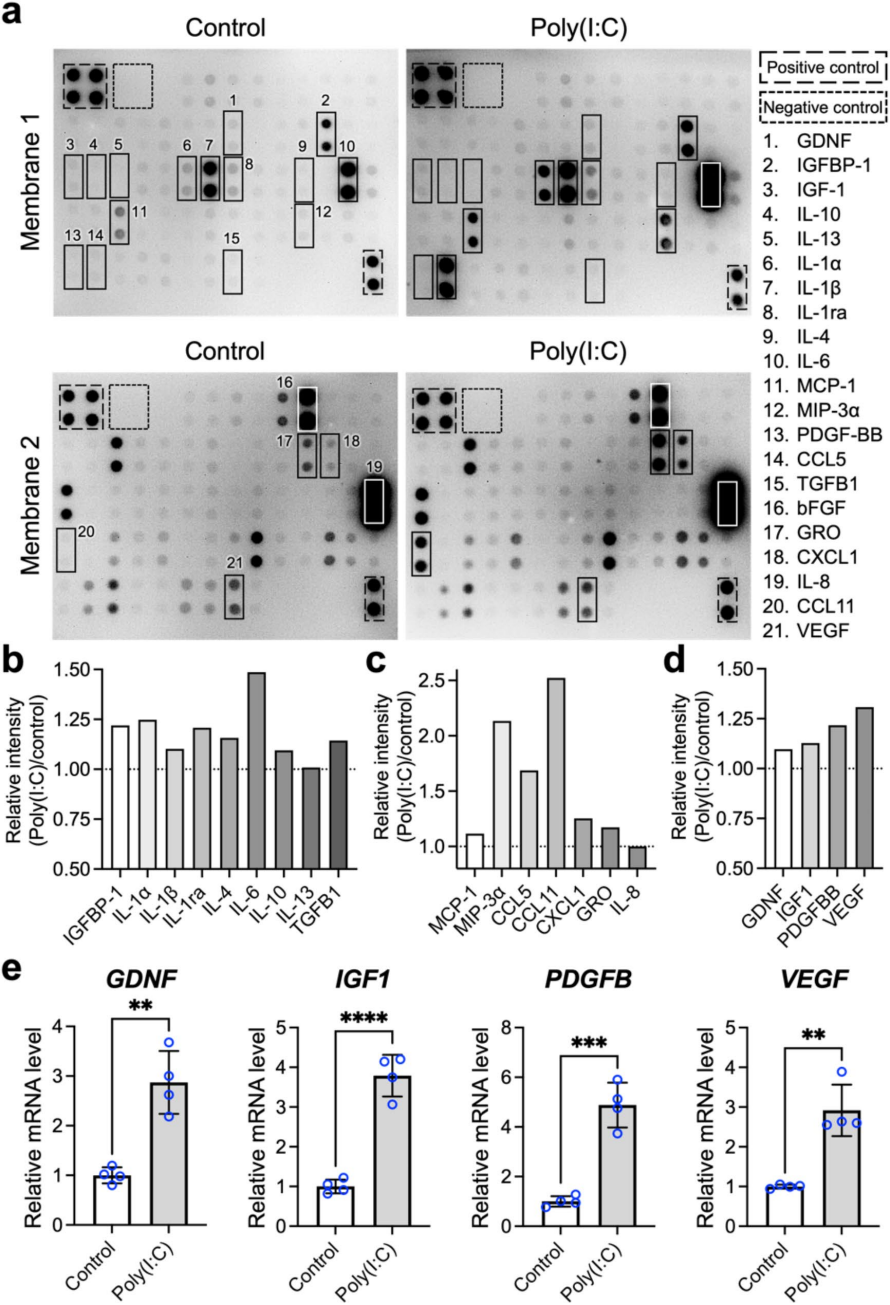
Poly(I:C) priming increase the paracrine activity of MSC spheroids. (**a**) Representative images of antibody array membranes from MSC spheroid-derived conditioned medium, with or without poly(I:C) treatment. The relative signal intensities of spots for (**b**) cytokines, (**c**) chemoattractants, and (**d**) growth factors in the poly(I:C)-treated group were normalized to those of the untreated control group. (**e**) mRNA expression levels of growth factors expressed by MSCs determined by qPCR (*n* = 4). The data are represented as the means ± SDs. Student’s *t* test was used to determine the *p* values. **p < 0.01; ***p < 0.005; ****p < 0.001

According to the images acquired from the antibody arrays (Fig. 3a) and their corresponding quantitative results, an increase in the levels of numerous cytokines (Fig. 3b) and chemoattractants (Fig. 3c) was observed in the CM derived from the poly(I:C)-primed MSC spheroids compared with that derived from the untreated MSC spheroids. Specifically, corroborating our qPCR results, the secretion of IL-1ra (1.21-fold), IL-4 (1.12-fold), and IL-10 (1.09-fold) by MSC spheroids was increased after poly(I-C) treatment (Fig. 3b). Furthermore, increased levels of IL-1α (1.25-fold), IL-1β (1.10-fold), IL-6 (1.49-fold), insulin like growth factor binding protein 1 (IGFBP1; 1.22-fold), and TGF-β1 (1.14-fold), which are all reported to be secreted by MSCs for exerting immunomodulatory functions, were found in CM derived from poly(I:C)-primed MSC spheroids (Fig. 3b). Moreover, poly(I:C) treatment further increased the release of multiple chemoattractants from MSC spheroids, including monocyte chemoattractant protein (MCP)-1 (1.12-fold), macrophage inflammatory protein (MIP)-3α (2.14-fold), C-C motif chemokine ligand (CCL) 5 (1.69-fold), CCL11 (2.52-fold), C-X-C motif ligand 1 (CXCL1; 1.25-fold), and growth-related oncogene (GRO; 1.17-fold; Fig. 3c). Collectively, these results reveal that poly(I:C) treatment promotes the expression of numerous enzymes and factors closely associated with the immunomodulatory capability of MSC spheroids, suggesting an enhanced potential for alleviating inflamed tissues.

MSCs rely on their paracrine activity not only for their ability to regulate immune responses but also for their potential to promote tissue regeneration. Therefore, we next focused on the impact of poly(I:C) priming on the growth factor content of the MSC spheroid-derived secretome. According to the results of the cytokine antibody arrays (Fig. 3a and d), the MSC spheroids treated with poly(I:C) presented elevated secretion of numerous growth factors, including glial cell line-derived neurotrophic factor (GDNF; 1.09-fold), insulin-like growth factor (IGF)-1 (1.13-fold), platelet-derived growth factor (PDGF)-BB (1.22-fold), and vascular endothelial growth factor (VEGF; 1.31-fold). These results were further validated through qPCR (Fig. 3e), which revealed poly(I:C)-induced upregulation of *GDNF* (2.87-fold; *p* < 0.01), *PDGFB* (4.88-fold; *p* < 0.005), *IGF1* (3.79-fold; *p* < 0.001), and *VEGFA* (2.92-fold; *p* < 0.01) expression. These findings suggest that poly(I:C) priming may increase not only the immunomodulatory potential of MSC spheroids but also their capacity to support tissue regeneration, making them highly promising for IBD treatment.

### Poly(I:C) priming increases the potential of MSC spheroids to induce Treg cells and suppress effector T (Teff) cells

Next, to explore the impact of poly(I:C)-induced alterations in the secretome profile of MSC spheroids on their potential to modulate T lymphocytes, which are highly associated with the progression of IBD, we collected CM from these spheroids and used it to treat primary mouse splenocytes, which contain various immune cells, including different T-cell populations. Flow cytometry analysis was then conducted to assess the effects of the MSC spheroid-derived CM on splenocytes.

**Fig. 4:**
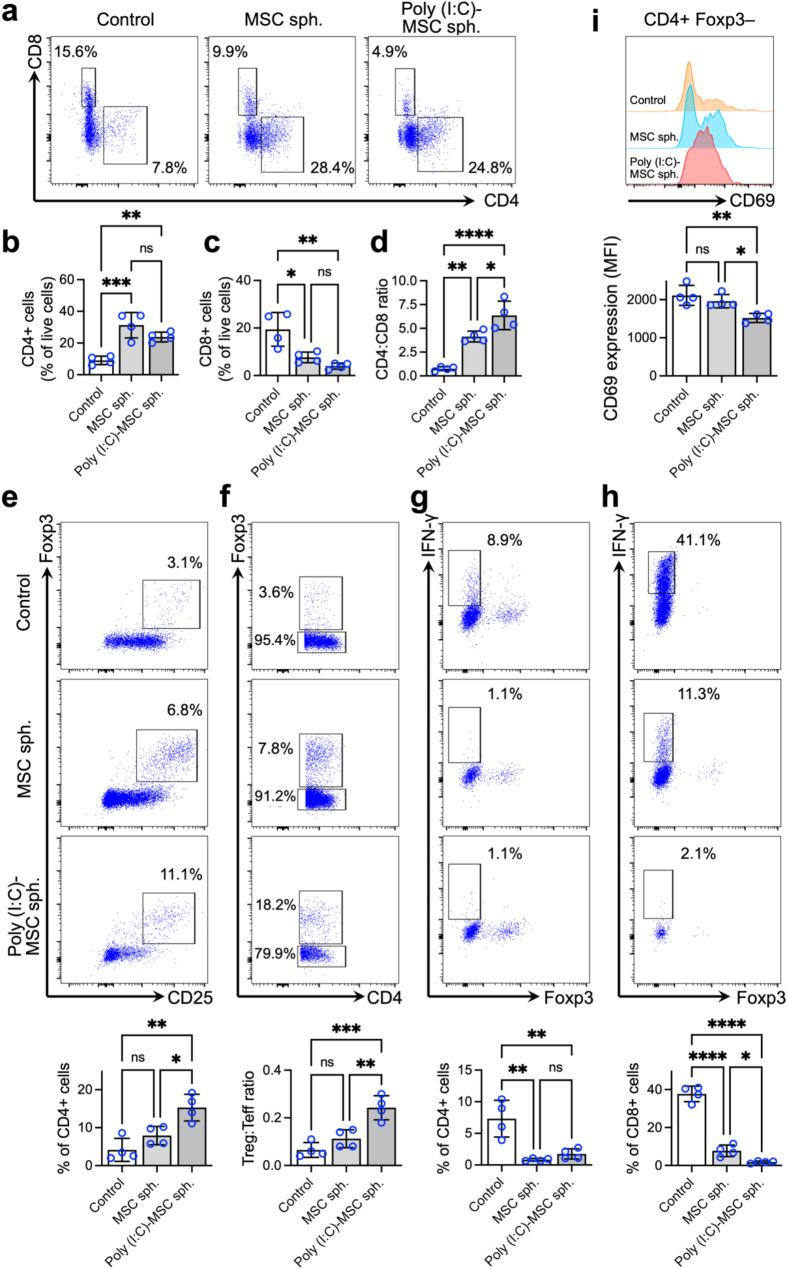
Poly(I: C) priming increases the potential of MSC spheroids to induce regulatory T (Treg) cells and suppress effector T (Teff) cells. (**a**) Representative flow cytometry plots and corresponding quantitative results of (**b**) CD4+ (*n* = 4), (**c**) CD8+ (*n* = 4) and (**d**) CD4-to-CD8 ratios (*n* = 4) in mouse splenocytes treated with MSC spheroid-derived CM. Representative flow cytometry plots and corresponding quantitative results of (**e**) the CD4+ CD25+ Foxp3+ population, (**f**) the CD4+ Foxp3+ (Treg) and CD4+ Foxp3– (Teff) population, (**g**) the CD4+ IFN-γ+ population, and (**h**) the CD8+ IFN-γ+ population in mouse splenocytes treated with MSC spheroid-derived CM (*n* = 4). (**i**) Representative histogram showing the frequency of CD69+ cells among CD4+ mouse splenocytes. The expression level of CD69 was determined by flow cytometry. MFI, mean fluorescence intensity. The data are represented as the mean ± SDs. ANOVA followed by Tukey’s test was used to determine the *p* values. *p < 0.05; **p < 0.01; ***p < 0.005; ****p < 0.001; ns, not significant

First, while the percentage of CD4+ T cells among the splenocytes in the untreated control group was approximately 9.1%, treatment with CM derived from MSC spheroids, without or with poly(I:C) priming, increased the percentage of CD4+ T cells to 31.3% (*p* < 0.005 vs. untreated control) and 23.9% (*p* < 0.01 vs. untreated control), respectively (Fig. 4a and b). Conversely, the percentage of CD8+ T cells in the untreated control group was 19.4%, which decreased to 7.6% (*p* < 0.05 vs. untreated control) and 3.9% (*p* < 0.01 vs. untreated control) after treatment with CM derived from MSC spheroids, without or with poly(I:C) priming, respectively (Fig. 4a and c). As a result, the CD4-to-CD8 ratios were significantly increased in splenocytes treated with MSC spheroid-derived CM (4.1 vs. 0.8 in the untreated control; *p* < 0.01) and were further increased in splenocytes treated with CM generated from poly(I:C)-primed MSC spheroids (6.4; *p* < 0.05 vs. CM from MSC spheroids without poly(I:C) priming; Fig. 4d).

In addition to the increased CD4-to-CD8 ratio, we observed a significantly increased percentage of CD4+ CD25+ Foxp3+ Treg cells in the poly(I:C)-primed MSC spheroid group (15.3% of CD4+ splenocytes) compared with that in the MSC spheroid group (7.9%; *p* < 0.05) and the untreated control group (4.2%; *p* < 0.01; Fig. 4e). Similarly, the ratio of Treg cells to Teff cells (CD4+ Foxp3–) was increased in splenocytes treated with poly(I:C)-primed MSC spheroid-derived CM (*p* < 0.01 vs. the MSC spheroid group; *p* < 0.005 vs. the untreated control group; Fig. 4f).

Furthermore, we investigated the frequency of interferon (IFN)-γ+ cells. Compared with the untreated control, both groups receiving CM derived from MSC spheroids, whether primed with poly(I:C) or not, presented a significant reduction in the percentages of CD4+ IFN-γ+ (Fig. 4g; *p* < 0.01 vs. untreated control) and CD8+ IFN-γ+ cells (Fig. 4h; *p* < 0.001 vs. untreated control). Moreover, CM derived from poly(I:C)-primed MSC spheroids exhibited a greater capacity to suppress CD8+ IFN-γ+ cells than CM derived from untreated MSC spheroids did (Fig. 4h; 1.8% vs. 7.8% *p* < 0.05). We also determined the CD4+ T-cell activation status by determining the mean fluorescence intensity of CD69. Compared with the untreated control, treatment with CM derived from MSC spheroids with poly(I:C) priming resulted in a significant reduction (Fig. 4i; *p* < 0.01). Taken together, these results indicate that MSC spheroids can suppress the Teff population and increase the Treg population. Furthermore, this immunomodulatory capacity can be significantly increased through poly(I:C) priming.

### Assembly into 3D spheroids and application of poly(I:C) synergistically increase the survival of MSCs following intraperitoneal transplantation into mice with IBD

Having verified the positive impact of poly(I:C) priming on the immunomodulatory capacity of MSC spheroids, we next examined whether poly(I:C) priming promotes the survival ability of MSCs in the presence of oxidative stress, a hostile microenvironment characteristic of IBD tissue [12]. Herein, we hypothesized that the assembly of MSCs into 3D spheroids combined with poly(I:C) priming could synergistically increase MSC viability under oxidative stress conditions. This hypothesis was preliminarily supported by our qPCR results (Fig. 2a), which indicated the upregulation of *HMOX1* expression, the gene encoding HO-1, an enzyme relevant to both the immunomodulatory capacity of MSCs [51] and cytoprotective mechanisms with antioxidative capacity [52]. To test this hypothesis, MSC suspensions or spheroids, with or without poly(I:C) priming, were collected and challenged with H_2_O_2_ to mimic oxidative stress when delivered into IBD tissue [3, 10]. The attachment of MSCs to the surface of the culture plate and their viability were assessed with a CCK-8 assay.

**Fig. 5:**
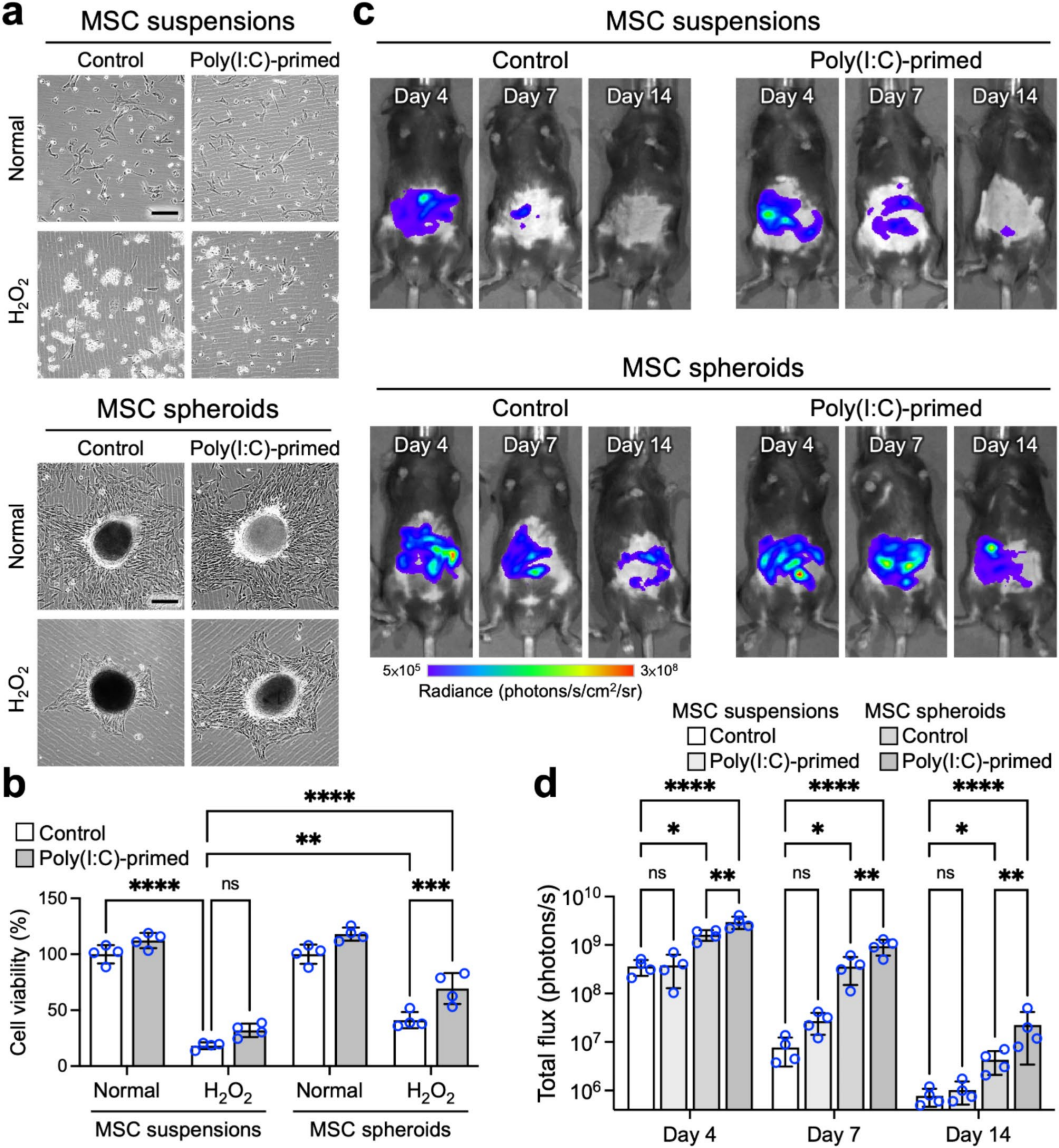
Assembly into 3D spheroids and application of poly(I:C) synergistically increase the survival of MSCs following intraperitoneal transplantation into mice with IBD. (**a**) Representative phase-contrast images of MSCs subjected to various treatments followed by exposure to oxidative stress and (**b**) corresponding results from the cell counting kit-8 (CCK-8) assay (*n* = 4). Scale bar, 300 μm. (**c**) Representative in vivo bioluminescence images of mice receiving MSC transplantation and (**d**) the corresponding bioluminescence intensities (*n* = 4 animals per group). The data are represented as the means ± SDs. ANOVA followed by Tukey’s test was used to determine the *p* values. *p < 0.05; **p < 0.01; ****p < 0.001; ns, not significant

For the MSC suspensions, exposure to H_2_O_2_ significantly impaired cellular attachment and viability (*p* < 0.001 vs. the normal control; Fig. 5a and b), and poly(I:C) priming provided only limited benefits, with no statistically significant increase in survival compared with that of the untreated MSC suspensions (32.1% vs. 18.1%; *p* > 0.05; Fig. 5a and b). In contrast, when assembled into 3D spheroids, MSCs exhibited approximately 2.3-fold greater viability under the same conditions (41.2%; *p* < 0.01; Fig. 5a and b), suggesting that the 3D spheroid configuration enables MSCs to better withstand elevated levels of oxidative stress. Furthermore, compared with unprimed MSC spheroids, poly(I:C)-primed MSC spheroids presented even greater survival rates (69.4%; 1.69-fold increase; *p* < 0.005; Fig. 5a and b). Collectively, these results demonstrate that assembling MSCs into 3D spheroids and priming them with poly(I:C) synergistically increases their survival under oxidative stress conditions (3.8-fold increase; *p* < 0.001; Fig. 5a and b), which may substantially improve post-engraftment cell viability.

Having explored the survival potential and immunomodulatory capability of poly(I:C)-primed MSC spheroids in vitro, we next examined the in vivo viability and therapeutic efficacy of these MSC spheroids in a DSS-induced mouse model of colitis. Following the initiation of DSS supplementation in drinking water, MSC suspensions or spheroids, with or without poly(I:C) priming, were administered twice (on day 1 and day 3) through intraperitoneal injection, with animals receiving saline treatment serving as the negative control.

Our first aim was to evaluate the survival of the delivered MSCs, which expressed NanoLuc and could be monitored in living animals with an in vivo imaging system (IVIS) [25]. The IVIS images (Fig. 5c) and associated bioluminescence measurements (Fig. 5d) revealed a sharp decrease in bioluminescence intensity in the cell suspension-treated groups, both with and without poly(I:C) priming, indicating substantial cell loss. Conversely, animals receiving MSCs in a 3D spheroid format presented significantly higher bioluminescent signal than those receiving MSC suspensions throughout the two-week experimental period did (*p* < 0.05; Fig. 5c and d), suggesting superior cell engraftment.

Furthermore, consistent with our in vitro results, MSC spheroids primed with poly(I:C) showed significantly greater cell viability than unprimed MSC spheroids did (*p* < 0.01; Fig. 5c and d). In summary, compared with the animals that received untreated MSC suspensions, the animals that received with poly(I:C)-primed MSC spheroids presented significantly greater bioluminescence (29.2-fold on day 14; *p* < 0.001; Fig. 5c and d), demonstrating that improved MSC engraftment was achieved through poly(I:C) treatment and spheroid formation.

Collectively, our in vitro and in vivo observations indicate that both the MSC assembly into 3D spheroids and poly(I:C) priming are effective strategies for increasing the post-engraftment survival of MSCs. Moreover, these strategies can be combined to achieve even greater improvements in cell viability.

### Poly(I:C)-primed MSC spheroids show enhanced efficacy in alleviating murine colitis

**Fig. 6:**
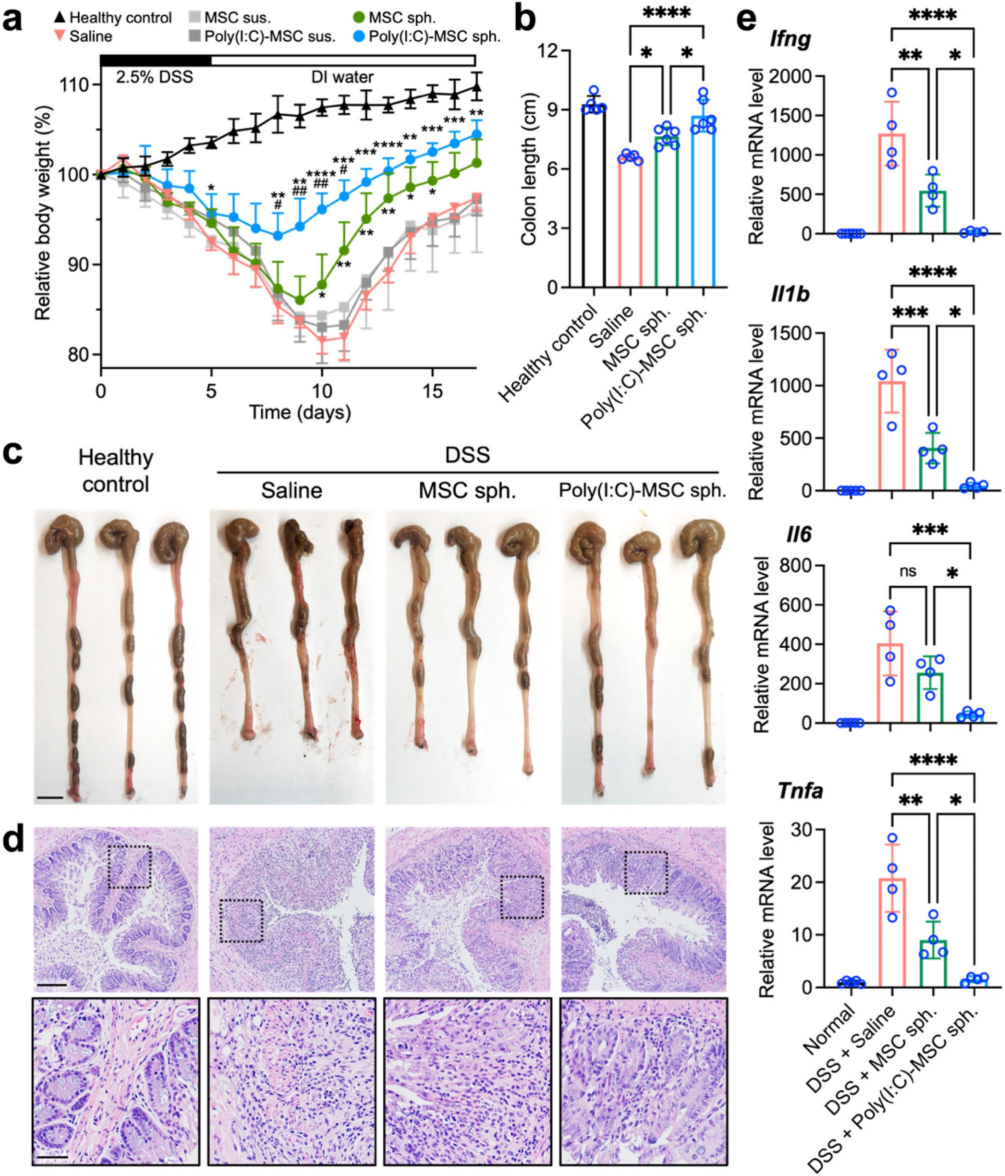
Poly(I:C)-primed MSC spheroids show increased efficacy in alleviating murine colitis. (**a**) Daily changes in the body weights of the test mice (*n* = 6 animals per group). *p < 0.05, **p < 0.01, ***p < 0.005, ****p < 0.001 vs. animals treated with saline; ^#^*p* < 0.05, ^##^*p* < 0.01 vs. animals treated with unprimed MSC spheroids. (**b**) Colon length of test mice harvested on day 9 (*n* = 6 animals per group). (**c**) Macroscopic appearance of the harvested colons. Scale bar, 1 cm. (**d**) Representative hematoxylin and eosin staining images of colon tissue. Scale bar, 200 μm (50 μm in zoomed-in panels). (**e**) Colonic mRNA expression levels of proinflammatory cytokines (*n* = 4 animals per group). *p < 0.05; **p < 0.01; ***p < 0.005; ****p < 0.001; ns, not significant. The data are represented as the means ± SDs. ANOVA followed by Tukey’s test was used to determine the *p* values

In addition to monitoring the survival of the engrafted MSCs, we simultaneously evaluated the therapeutic effect by tracking the body weights of the mice with colitis. During the 5-day period of DSS addition to their drinking water, the test animals exhibited a significant decrease in body weight (Fig. 6a). In the control group, which received saline, the body weight continued to decrease until day 10, even after DSS supplementation stopped on day 5, after which body weight gradually increased. By day 17, their body weight had recovered to 97.4% of the initial weight.

The intraperitoneal injection of MSC suspensions, whether or not they were poly(I:C)-primed, did not result in significant therapeutic benefits, as the body weight change trends for both groups were similar to those of the saline control group (Fig. 6a). In contrast, the transplantation of MSC spheroids effectively mitigated body weight loss in mice compared to those receiving MSC suspensions or saline (Fig. 6a; *p* < 0.05 from day 10 to day 15 vs. saline group). Notably, poly(I:C) priming significantly increased the therapeutic potential of the MSC spheroids (Fig. 6a; *p* < 0.01 from day 8 to day 17 vs. the saline group; *p* < 0.05 from day 8 to day 11 vs. the unprimed MSC spheroid group). In the animals treated with poly(I:C)-primed MSC spheroids, the body weight only decreased to 93.2% of the initial value after the onset of DSS-induced IBD, whereas it was 86.1% in the animals that received unprimed MSC spheroids. In summary, MSCs delivered as 3D spheroids were more effective at alleviating IBD than those transplanted as single-cell suspensions were, and poly(I:C) priming further improved the therapeutic outcomes of MSC spheroids.

To gain insight into the therapeutic effect of MSC spheroid transplantation, we harvested the colons 9 days after the beginning of DSS supplementation. Our results indicated that, in addition to alleviating body weight loss, animals that underwent MSC spheroid transplantation showed reduced colon shortening and improved stool consistency (6.6 ± 0.4 cm in the saline group vs. 7.7 ± 0.5 cm in the MSC spheroid group; *p* < 0.05; Fig. 6b and c). Furthermore, compared with unprimed MSC spheroids, poly(I:C)-primed MSC spheroids had a superior therapeutic effect on reversing DSS-induced colon shortening (8.7 ± 0.8 cm; *p* < 0.001 vs. the saline group; *p* < 0.05 vs. the unprimed MSC spheroid group; Fig. 6b and c). Histologically, the transplantation of poly(I:C)-primed MSC spheroids was more effective at reducing epithelial disruption and ameliorating granulocyte infiltration than the transplantation of unprimed MSC spheroids (Fig. 6d).

We also analyzed the mRNA expression levels of proinflammatory cytokines in colon tissues. Compared with those in the saline group, the expression level of *Ifng*, *Il1b* and *Tnfa* was significantly lower in the MSC spheroid group (Fig. 6e; *p* < 0.01), suggesting reduced inflammation. The level of *Il6* mRNA expression also decreased, although the difference was not statistically significant (Fig. 6e). Moreover, the transplantation of poly(I:C)-primed MSC spheroids led to a more significant downregulation of *Ifng*, *Il1b*, *Il6* and *Tnfa* than did the transplantation of unprimed MSC spheroids (Fig. 6e; *p* < 0.05). Collectively, our in vivo results demonstrate that the therapeutic potential of MSC spheroids for alleviating IBD can be effectively increased through poly(I:C) priming.

## Discussion

The application of MSCs for alleviating IBD has been hampered by heterogeneous efficacy. Conventional MSC-based therapies typically administer single-cell suspensions, either systemically or locally, but these approaches suffer from poor engraftment due to rapid clearance, limited retention at inflamed sites, and reduced functionality in adverse microenvironments [12, 16, 17, 19–23]. In contrast, the transplantation of MSCs as 3D spheroids has been shown to overcome these limitations and enhance MSC-mediated tissue regeneration in various inflammation-related disease models, including ischemic stroke [25], myocardial infarction [53], lung injury [54], kidney dysfunction [28], tendon rupture [29], and liver failure [55].

In this study, we developed a combination approach that integrates both the structural benefits of preassembled 3D spheroids and the immunomodulatory enhancement of TLR3 activation *via* poly(I:C) priming, aiming to improve MSC survival in the hostile IBD microenvironment and enhance their immunomodulatory potential. Impressively, the assembly of MSCs into 3D spheroids induced TLR3 expression upregulation, further amplifying the effect of poly(I:C)-driven TLR3 activation and increasing their immunomodulatory capacity. Our in vitro analysis confirmed that this combination approach increased the secretion of numerous cytokines and growth factors relevant to the immunomodulatory and pro-regenerative potential of MSCs and enhanced resistance to oxidative stress.

Compared with conventional MSC suspensions, spheroid-based MSC delivery demonstrated superior retention, improved survival, and greater therapeutic efficacy in alleviating colon inflammation and preventing colonic shortening in mice with DSS-induced IBD, with poly(I:C) priming further amplifying these effects. By simultaneously addressing key challenges in MSC transplantation, our approach significantly improved therapeutic outcomes. These findings highlight the potential of this combination strategy as a promising therapeutic approach to increase the consistency and efficacy of MSC-based therapies, not only for IBD but also for a broader range of inflammation-related diseases.

TLR3-mediated signaling pathways are crucial to the immunomodulatory potential of MSCs [16, 39, 56]. Previous studies showed that the upregulation of TLR3 expression in MSCs occurs primarily in an autocrine manner, where stimulation with TLR3 ligands increases TLR3 expression in MSCs [56, 57]. To our knowledge, cell assembly-induced upregulation of TLR3 expression, as discovered in the present study, has not been previously reported. Although the formation of 3D spheroids has been shown to alter the secretion profile of MSCs and various intracellular signaling pathways, potentially contributing to increased TLR3 expression levels, further investigations are needed to elucidate the detailed mechanisms underlying the observed TLR3 expression upregulation upon spheroid formation.

Our study demonstrated that MSCs grown as spheroids exhibit increased immunomodulatory potential, as reported by us [30] and others [31, 34]. This self-activation was evident through a significant increase in the expression levels of COX-2 and IDO1, along with increased secretion of PGE2, in the control MSC spheroids compared with that in the cells grown as monolayers, even without poly(I:C) supplementation. Furthermore, poly(I:C) priming significantly upregulated the expression of numerous enzymes and factors associated with the immunomodulatory capacity of MSC spheroids, a phenomenon previously observed only in MSCs grown as 2D monolayers [58, 59]. These findings were corroborated by our antibody array data, which indicated a substantial increase in the secretion of various cytokines and chemokines in poly(I:C)-primed MSC spheroids, consistent with reports in monolayer-cultivated MSCs [38, 58, 60–63]. In addition to the cytokines that can directly regulate immune cell behaviors, the increased release of chemoattractants observed in our study likely contributes significantly to the enhanced immunomodulatory effect of MSC spheroids, given that the migration and recruitment of immune cells to the vicinity of MSCs is crucial for establishing immunomodulation [60]. Further studies are warranted to elucidate the detailed mechanisms behind these observations and to explore the impact of poly(I:C) priming on immune cell recruitment by MSC spheroids, improving their potential for use in clinical applications.

In addition to modulating inflammatory responses, another potential therapeutic mechanism of MSCs for IBD treatment is the release of growth factors that suppress intestinal epithelial cell death, promote tissue repair, and thereby favor cellular homeostasis and intestinal integrity [64, 65]. For example, GDNF has been reported to exert direct effects on the intestinal epithelial barrier by promoting barrier maturation and wound healing [66, 67]. Similarly, IGF-1 has the potential to attenuate intestinal damage and promote mucosal repair in colitis [68]. VEGF-mediated angiogenesis, although involved in the pathogenesis of IBD, plays a major role in intestinal epithelial wound healing [69, 70]. Our antibody array data revealed increased secretion of these growth factors by MSC spheroids upon poly(I:C) priming, which may also contribute to the enhanced therapeutic efficacy of MSC spheroids.

In addition to the increased immunomodulatory and pro-regenerative potential, our results indicated that the formation of 3D spheroids and poly(I:C) priming synergistically promoted MSC survival under hostile conditions, such as oxidative stress. Consistent with previous studies demonstrating that 3D spheroid assembly [71] and poly(I:C) treatment [72] individually promote the expression of HO-1, a cytoprotective enzyme crucial for the cellular response to oxidative stress [52], our data further indicated that HO-1 levels could be significantly increased by priming MSC spheroids with poly(I:C). This enhancement might result in increased survival of MSC spheroids under in vitro H_2_O_2_ challenge and in vivo IBD models, presumably leading to better therapeutic outcomes, including alleviated body weight reduction, reduced colon shortening, and decreased immune cell infiltration and proinflammatory cytokine expression.

Despite the observed increase in cell viability, a significant and continuous reduction in the bioluminescence signal intensity emitted from poly(I:C)-primed MSC spheroids was found within the two-week follow-up period after intraperitoneal transplantation, indicating ongoing cell loss. Although further strategies to prolong MSC survival can be developed, the relatively short-lived nature of MSCs can also contribute to their safety profile [73]. Therefore, our approach only transiently prolonged the viability of MSCs for IBD treatment without increasing their presence in the body, thus potentially reducing safety concerns for future clinical applications.

Although this study has advanced the development of more effective MSC-based therapies for IBD by providing an efficient approach to improve cell viability and therapeutic potential, several limitations must be addressed before clinical translation. First, the current investigation was focused on the impact of poly(I:C)-mediated TLR3 activation on MSC spheroids in terms of the expression of immunomodulatory molecules and growth factors. However, TLR3 signaling pathways are known to influence various cellular behaviors, such as migration, proliferation, and metabolism, which can affect the therapeutic functionality of MSCs beyond their paracrine secretome. Consequently, comprehensive analyses exploring changes in mRNA expression and protein levels are necessary to elucidate the overall impact of poly(I:C) priming on MSC spheroids.

Furthermore, while our results demonstrated that poly(I:C) priming significantly increased the regulatory potential of MSC spheroids on mouse splenocytes, additional investigations into the in vivo changes in T-cell populations and their behaviors in the spleen and mesenteric lymph nodes of the mouse IBD model will provide deeper insights into the immunomodulatory effects of poly(I:C)-primed MSC spheroids. Additionally, spatial analyses of engrafted poly(I:C)-primed MSC spheroids, immune cell populations, and key proinflammatory cytokines in colon tissues could help reveal the localized immune landscape shaped by these transplanted cells. Expanding on these aspects in future studies will be crucial for fully characterizing the therapeutic potential of this approach and optimizing its application in inflammatory diseases.

Moreover, human-sourced MSCs were utilized in this study, and although these cells are generally considered to establish an immune-privileged microenvironment through their immunomodulatory functions, it is critical to investigate whether xenograft-induced host immune reactions contribute to the observed therapeutic outcomes. For example, the rapid loss of intraperitoneally transplanted cells observed in the group that received MSC suspensions may have led to the ineffectiveness in ameliorating mouse colitis. Further research is needed to determine whether this rapid cell loss is attributable to xenograft-induced rejection.

Finally, despite the promising effects of poly(I:C) priming on MSC spheroids, an approach extensively studied in 2D MSC cultures [38–40], its clinical application remains limited, as it is confined primarily to research settings. Key concerns include differences in stability and efficacy among poly(I:C) formulations (naked, sodium and potassium salts, or polylysine and carboxymethylcellulose-stabilized forms) [74, 75], residual poly(I:C) within MSC spheroids prior to transplantation, and potential immunogenicity or off-target effects post-transplantation. Further investigation is essential to optimize safety, minimize adverse effects, and facilitate the clinical translation of this strategy.

## Conclusions

In conclusion, we demonstrated that poly(I:C)-driven TLR3 activation is effective for the MSCs grown as 3D spheroids and can be employed as a combinational approach to increase the post-engraftment survival of MSCs, along with their immunomodulatory and pro-regenerative potential for IBD treatment. The findings of this research not only increase the therapeutic potential of MSCs for IBD but also pave the way for MSC application in a wider array of therapeutic contexts, potentially revolutionizing the treatment of inflammation-related diseases.

## Electronic supplementary material

Below is the link to the electronic supplementary material.

Supplementary Material 1

## Data Availability

The raw/processed data required to reproduce these findings are available from the corresponding authors upon reasonable request.
