# Supplementary material for: Assembly of MSCs into a spheroid configuration increases poly(I:C)-mediated TLR3 activation and the immunomodulatory potential of MSCs for alleviating murine colitis
Source: Stem Cell Res Ther. 2025 Apr 12;16:172. doi: 10.1186/s13287-025-04297-3 (PMC11993957; doi:10.1186/s13287-025-04297-3)
Supplement: Supplementary file 1 — Supplementary Material 1 [file 13287_2025_4297_MOESM1_ESM.docx]

**Table S1**. Primer sequence used for real-time quantitative polymerase chain reaction.

| **Species** | **Gene** | **Forward** | **Reverse** |
| --- | --- | --- | --- |
| Human | *BDNF* | 5’-GTCAAGTTGGGAGCCTGAAATAGTG-3’ | 5’-AGGATGCTGGTCCAAGTGGTG-3’ |
|  | *HMOX1* | 5’-AACTTTCAGAAGGGCCAGGT-3’ | 5’-CTGGGCTCTCCTTGTTGC-3’ |
|  | *IDO1* | 5’-GCCCTTCAAGTGTTTCACCAA-3’ | 5’-GCCTTTCCAGCCAGACAAATAT-3’ |
|  | *IGF1* | 5’-GGTGGATGCTCTTCAGTTCGTG-3’ | 5’-AAATGTACTTCCTTCTGGGTCTTGG-3’ |
|  | *IL1RN* | 5’-AAGATGTGCCTGTCCTGTGTCAA-3’ | 5’-GTTCTCGCTCAGGTCAGTGATGTTA-3’ |
|  | *IL4* | 5’-CCGTAACAGACATCTTTGCTGCC-3’ | 5’-GAGTGTCCTTCTCATGGTGGCT-3’ |
|  | *IL10* | 5’-GACTTTAAGGGTTACCTGGGTTG-3’ | 5’-TCACATGCGCCTTGATGTCTG-3’ |
|  | *PDGFB* | 5’-GGCCGAGTTGGACCTGAACATGA-3’ | 5’-GAAGTTGGCGTTGGTGCGGTCTA-3’ |
|  | *PTGS2* | 5’-GAATGGGGTGATGAGCAGTT-3’ | 5’-CAGAAGGGCAGGATACAGC-3’ |
|  | *RPL13A* | 5’-CATAGGAAGCTGGGAGCAAG-3’ | 5’-GCCCTCCAATCAGTCTTCTG-3’ |
|  | *TGFB1* | 5’-CCCAGCATCTGCAAAGCTC-3’ | 5’-GTCAATGTACAGCTGCCGCA-3’ |
|  | *TLR3* | 5’-TCAACACTGTTATGTTTGTGGGT-3’ | 5’-TTGCCTTGTATCTACTTTTGGGG-3’ |
|  | *TNFAIP6* | 5’-GATGGATGGCTAAGGGCAGAGT-3’ | 5’-TCATTTGGGAAGCCTGGAGATT-3’ |
|  | *VEGFA* | 5’-TCTTCAAGCCATCCTGTGTG-3’ | 5’-ATCCGCATAATCTGCATGGT-3’ |
| Mouse | *Gapdh* | 5’-CTGCCACCCAGAAGACTGTG-3’ | 5’-GGTCCTCAGTGTAGCCCAAG-3’ |
|  | *Ifng* | 5’-GATGCATTCATGAGTATTGCCAAGT-3’ | 5’-GTGGACCACTCGGATGAGCTC-3’ |
|  | *Il1b* | 5’-GCCCATCCTCTGTGACTCAT-3’ | 5’-AGGCCACAGGTATTTTGTCG-3’ |
|  | *Il6* | 5’-TCCAGTTGCCTTCTTGGGAC-3’ | 5’-GTGTAATTAAGCCTCCGACTTG-3’ |
|  | *Tnfa* | 5’-CCGATGGGTTGTACCTTGT-3’ | 5’-CGGACTCCGCAAAGTCTAAG-3’ |


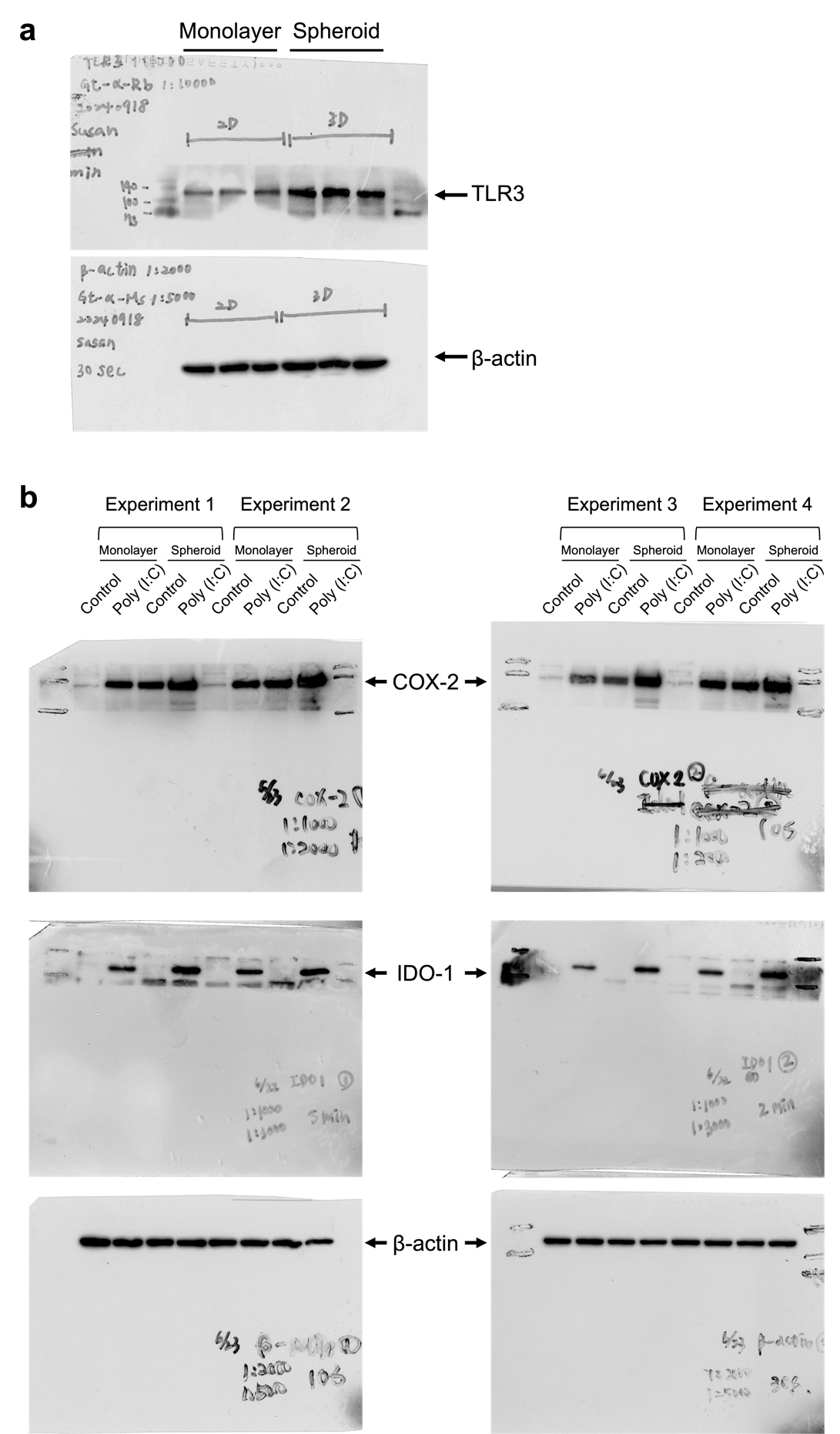


**Supplementary Figure 1. Uncropped gels of all proteins analyzed in Western blot experiments.** (a) Uncropped gels for TLR3 and β-actin expression in MSCs corresponding to Figure 1b. (b) Uncropped gels for COX-2, IDO-1, and β-actin expression in MSCs corresponding to Figure 2b. Areas outlined by dotted lines are cropped and presented in Figure 2b.
